# Giant cell arteritis exclusively detected by ^18^F-fluorodeoxyglucose positron emission tomography: a case report

**DOI:** 10.1186/1752-1947-8-356

**Published:** 2014-10-28

**Authors:** Markus Brückner, Dominik Bettenworth, Karin Hengst, Matthias Weckesser, Peter Willeke, Jan Heidemann

**Affiliations:** 1 Department of Medicine B, University of Münster, Albert-Schweitzer-Campus 1, D-48129 Münster, Germany; 2 Department of Nuclear Medicine, University of Münster, Albert-Schweitzer-Campus 1, D-48129 Münster, Germany; 3 Department of Medicine D, University of Münster, Albert-Schweitzer-Campus 1, D-48129 Münster, Germany; 4 Klinikum Bielefeld, Department of Gastroenterology, Teutoburger Str. 50, D-33604 Bielefeld, Germany

**Keywords:** Fever of unknown origin, ^18^F-FDG PET, ^18^F-fluorodeoxyglucose positron emission tomography, Giant cell arteritis, Large vessel vasculitis

## Abstract

**Introduction:**

This case of giant cell arteritis is noteworthy because it evaded standard diagnostic criteria and only emerged as fever of unknown origin. In this regard, we present ^18^F-fluorodeoxyglucose positron emission tomography as a valid diagnostic method.

**Case presentation:**

This case report describes a 58-year-old Caucasian woman who is a cigarette smoker with a 10-week history of fever of unknown origin, night sweats and weight loss of 12kg. Initially, clinical presentation was suspicious of malignant disease. Laboratory findings detected significantly elevated inflammatory blood parameters including C-reactive protein and elevated erythrocyte sedimentation rate (110mm/hour). Extensive diagnostic workup including microbiological and rheumatological assessment, ultrasonography, endoscopy and computed tomography of abdomen and thorax did not indicate any septic or malignant focus. Eventually, ^18^F-fluorodeoxyglucose positron emission tomography was able to reveal arteritis of her aortic arch and supraaortic branches. Subsequently, she commenced steroid and methotrexate therapy that led to sustained remission.

**Conclusions:**

This case of giant cell arteritis may promote discussion regarding a more specific classification for this disease entity. Furthermore, it confirms that ^18^F-fluorodeoxyglucose positron emission tomography might serve as a valuable tool for diagnosis of giant cell arteritis, because it could facilitate an accurate and non-invasive detection of lesions of large vessels.

## Introduction

Giant cell arteritis (GCA) is the most common rheumatological vasculopathy in Caucasians in Europe [1]. Clinical findings comprise new onset of temporal headache, jaw claudication, visual loss, temporal artery induration, thoracic aortic aneurysm or intense pain in pectoral and pelvic girdle with morning stiffness [2]. In addition, systemic symptoms including fatigue, weight loss or fever occur in up to 50%. Furthermore, GCA may be the cause of fever of unknown origin (FUO) in up to 17% of patients with FUO above 50 years of age [3,4]. A retrospective study of 100 patients with GCA found fever to exceed 39°C in 15% [5]; fever can be the single presenting symptom.

In contrast to common presentations of GCA, we describe an exceptional clinical course presenting with FUO as the only clinical feature in the absence of other usual clinical characteristics.

## Case presentation

A 58-year-old Caucasian obese woman (body mass index 31.5kg/m^2^) was admitted to our hospital with a history of continuous FUO varying from 38°C to 39.5°C for 10 weeks despite several courses of antibiotic therapy. Her history included 30 pack years of cigarette smoking. Currently, she suffered from night sweats, continuous weight loss of 12kg and dyspnea on exertion. No signs of rheumatic or neurological diseases were present, especially no temporal pain, headache or visual impairment. There were no skin lesions, no myalgia of pectoral or pelvic girdle or morning stiffness, and no arthralgia. Her blood pressure was 140/90mmHg on both arms. Initial suspected diagnosis involved malignant or infectious diseases. Laboratory findings showed elevated systemic parameters of inflammation including C-reactive protein (CRP; 22mg/dL) and accelerated erythrocyte sedimentation rate (ESR; 110mm/hour). Complete blood count showed total white blood cell 13.8/μL, total red blood cell 5 million/μL, hemoglobin 13.4g/dL, hematocrit 42.8%, mean corpuscular volume 84.4fL, mean corpuscular hemoglobin 26.4pg, mean corpuscular hemoglobin concentration 31.3g/dL, and platelet 278,000/μL. Differential blood count showed neutrophil granulocytes 88.1%, lymphocytes 8.1%, monocytes 3.6%, eosinophil granulocytes 0.1%, and basophil granulocytes 0.5%. Mean platelet volume was 10.4fL. Serum albumin showed 32.8g/L and β2 microglobulin 1.9mg/L. Detailed blood tests for rheumatic disorders were negative. Repeated microbiological blood tests, stool cultures as well as serological testing for specific pathogens, including *Orthomyxoviridae, Herpesviridae, Mycobacteriaceae, Legionellaceae, Spirochaetaceae, Chlamydiaceae* and *Mycoplasmataceae* were negative.

**Figure 1:**
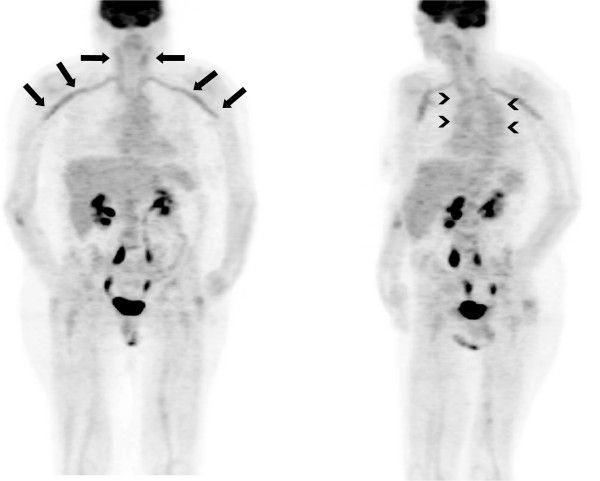
^**18**^**F-fluorodeoxyglucose positron emission tomography whole-body image.** In particular, subclavian arteries (arrows) show increased metabolic activity (maximal standardized uptake value 3.0, mean liver standardized uptake value 1.8). The oblique projection shows increased glucose metabolism in the aortic arch (arrow heads) too. No other foci of increased metabolism like solid tumors were detectable in this imaging.

Endoscopic evaluations, including bronchoscopy, esophagogastroduodenoscopy and colonoscopy as well as transesophageal echocardiography were without pathological findings. In addition, computed tomography (CT) of her thorax and abdomen showed atherosclerotic lesions only; however, no evidence of any inflammatory or malignant focus was detected. In particular, there were no alterations of the vessel wall indicative of inflammation. We decided to perform ^18^F-fluorodeoxyglucose positron emission tomography (^18^F-FDG PET; Figure 1). ^18^F-FDG PET demonstrated increased metabolic activity in her supraaortic arteries with the most intense involvement of the subclavian arteries and also slightly elevated glucose metabolism in the aortic arch. A borderline asymmetry of the femoral artery was regarded as within physiological limits. There were no additional foci of increased glucose metabolism in her body. Thus, GCA of the supraaortic arteries was proposed as the inflammatory focus. Other causes for aortitis or arteritis (Gsell-Erdheim disease, syphilitic aortitis) and FUO had been excluded initially. After commencement of steroid therapy with 90mg prednisolone daily intravenously, her inflammatory markers dropped rapidly within 10 days (CRP 6mg/dL) and she was discharged in good health. Over a period of 6 weeks steroids were reduced to the lowest effective dose of 40mg daily orally with parameters of disease being clinical symptoms, CRP, and ESR. Methotrexate was added at a dose of 20mg weekly subcutaneous injection to support steroid tapering. Today, she consults our rheumatologic out-patient clinic at intervals of 6 months, the medication has been reduced to 2mg oral prednisolone daily and methotrexate 20mg weekly subcutaneous injection.

## Discussion

Our case clearly shows the limitations of 1990 American College of Rheumatology criteria for GCA [6]: age over 50 years, new onset of localized headache, temporal artery tenderness, elevated ESR for more than 50mm/hour and biopsy sample showing necrotizing arteritis with giant cells. Weyand and Goronzy [7] report blindness, headache and jaw claudication as the classical clinical symptoms in cranial arteritis. However, rather systemic characteristics of GCA such as night sweats, loss of weight, and in particular FUO can be the only presenting symptoms, highlighting the necessity of a more specific classification. Weyand *et al*. [8,9] suggested clinical subtypes of GCA affecting distinct vascular territories with different patterns of cytokine production, leading to occlusive or non-occlusive disease and different features in temporal artery biopsy. These subtypes are cranial arteritis, large vessel arteritis or aortitis, isolated polymyalgia rheumatica and, as depicted in the present case, systemic inflammatory syndrome with arteritis.

If GCA presents as systemic inflammatory syndrome only, then FUO diagnostics provoke high costs for diagnostic management and duration of hospitalization [10,11]. As the causes for FUO are numerous and heterogeneous, there is no gold standard in the diagnostic process. Since FUO is well known to be a possible symptom in patients with GCA, temporal artery biopsy is crucial in the diagnostic workup after initial negative findings [12]. Biopsy is still recommended as the gold standard as a sophisticated procedure in local anesthesia with low complication rates; however, only 50% of biopsy samples show multinucleated giant cells with even 10 to 25% false negative results [13]. Moreover, even the combination of FUO and asymptomatic thoracic manifestations such as pleural effusions may be presenting symptoms of GCA [14]. Because of its clinical heterogeneity, initial diagnosis of GCA and evaluation of its extent remain challenging, leading to specific advantages and disadvantages of conventional imaging procedures such as CT and magnetic resonance imaging [15]. In obscure cases like ours, nuclear imaging as a second line of diagnostic provides high standards concerning sensitivity and specificity [16,17] helping to identify the cause of fever. In our patient, inflammation of the aortic arch and subclavian arteries could only be visualized by ^18^F-FDG PET.

Recently, it could be shown that ^18^F-FDG PET provides a high level of effectiveness in assessing initial diagnosis, extent and activity of GCA [18,19]. Other nuclear methods such as gadolinium scanning are also available for detection of vascular wall inflammation, although they possess less sensitivity and specificity. However, there are several limitations of ^18^F-FDG PET, which cannot reliably be used to diagnose or monitor inflammation of the temporal artery due to the limited spatial resolution of PET alone; the combination of PET and CT (PET-CT) generally has superior spatial resolution. For this reason, PET cannot replace temporal artery biopsy if the disease is limited to the temporal arteries [20]. Finally, atherosclerosis as the most frequent inflammatory disease of the arteries is the most important differential diagnostic feature of increased tracer uptake in the blood vessels.

## Conclusions

In the case presented here, classical clinical characteristics of GCA were missing, while FUO was the leading symptom. Finally, our case proves that ^18^F-FDG PET is able to serve as a valuable tool for diagnosis of GCA. ^18^F-FDG PET can be a valid diagnostic method when manifestations such as vascular insufficiency are lacking. In addition, new criteria for the classification of GCA are needed to detect this disease at early stage.

## Consent

Written informed consent was obtained from the patient for publication of this case report and accompanying images. A copy of the written consent is available for review by the Editor-in-Chief of this journal.

## Abbreviations

CRP: C-reactive protein; CT: Computed tomography; ESR: Erythrocyte sedimentation rate; ^18^F-FDG PET: ^18^F-fluorodeoxyglucose positron emission tomography; FUO: Fever of unknown origin; GCA: Giant cell arteritis.

## Competing interests

The authors declare that they have no competing interests.

## Authors’ contributions

MB and DB designed and wrote the paper and took care of the patient. MW analyzed and provided images of ^18^F-FDG PET and revised the paper as a senior physician for nuclear medicine. PW wrote the paper, revised it as a senior physician for rheumatology and took care of the patient. JH and KH wrote the paper, revised it as senior physicians for internal medicine and took care of the patient. All authors read and approved the final manuscript.
